# Development of a 3-dimensional organotypic model with characteristics of peripheral sensory nerves

**DOI:** 10.1016/j.crmeth.2024.100835

**Published:** 2024-08-07

**Authors:** Madoka Koyanagi, Ryosuke Ogido, Akari Moriya, Mamiko Saigo, Satoshi Ihida, Tomoko Teranishi, Jiro Kawada, Tatsuya Katsuno, Kazuo Matsubara, Tomohiro Terada, Akira Yamashita, Satoshi Imai

**Affiliations:** 1 Department of Medical Neuropharmacology, School of Pharmaceutical Sciences, Wakayama Medical University, Wakayama 640-8156, Japan; 2 Department of Clinical Pharmacology and Therapeutics, Graduate School of Pharmaceutical Sciences, Kyoto University, Kyoto 606-8507, Japan; 3 Department of Clinical Pharmacology and Therapeutics, Faculty of Pharmaceutical Sciences, Kyoto University, Kyoto 606-8507, Japan; 4 Department of Clinical Pharmacology and Therapeutics, Kyoto University Hospital, Kyoto 606-8507, Japan; 5 New Business Promotion Division, Business Development Unit, Panel Semicon Laboratories, Sharp Corporation, Tenri, Nara 632-8567, Japan; 6 Jiksak Bioengineering, Inc., Kawasaki, Kanagawa 210-0821, Japan; 7 Division of Electron Microscopic Study, Center for Anatomical Studies, Graduate School of Medicine, Kyoto University, Kyoto 606-8501, Japan; 8 School of Pharmaceutical Sciences, Wakayama Medical University, Wakayama 640-8156, Japan

**Keywords:** organotypic model, ex vivo explant culture, microfluidic device, sensory nerve, peripheral nervous system, Schwann cells, myelin, node of Ranvier, peripheral neuropathy, nerve regeneration

## Abstract

We developed a rat dorsal root ganglion (DRG)-derived sensory nerve organotypic model by culturing DRG explants on an organoid culture device. With this method, a large number of organotypic cultures can be produced simultaneously with high reproducibility simply by seeding DRG explants derived from rat embryos. Unlike previous DRG explant models, this organotypic model consists of a ganglion and an axon bundle with myelinated A fibers, unmyelinated C fibers, and stereo-myelin-forming nodes of Ranvier. The model also exhibits Ca^2+^ signaling in cell bodies in response to application of chemical stimuli to nerve terminals. Further, axonal transection increases the activating transcription factor 3 mRNA level in ganglia. Axons and myelin are shown to regenerate 14 days following transection. Our sensory organotypic model enables analysis of neuronal excitability in response to pain stimuli and tracking of morphological changes in the axon bundle over weeks.

## Highlights

• A rat dorsal root ganglion (DRG)-derived sensory nerve organotypic model is generated
• The rat DRG explants are cultured on an organoid culture device
• The model consists of myelinated A fibers, unmyelinated C fibers, and nodes of Ranvier
• It recapitulates characteristics of the peripheral nervous system

## Motivation

Although existing *in vitro* and *ex vivo* approaches to research the peripheral nervous system (PNS) have advantages, it remains challenging to recapitulate peripheral nerves with respect to structural or technical aspects. In fact, several previous sensory nerve organotypic models have not been demonstrated to contain cell populations that constitute the PNS and have not been optimized to explore the complex machinery underlying peripheral neuropathy. Therefore, an experimental platform is necessary to investigate reciprocal communications between sensory neurons and myelinating Schwann cells in the PNS and temporally track them. Here, we developed a rat dorsal root ganglion (DRG)-derived sensory nerve organotypic culture that is structurally more similar than other models to peripheral nerves.

## Graphical abstract

**Figure:**
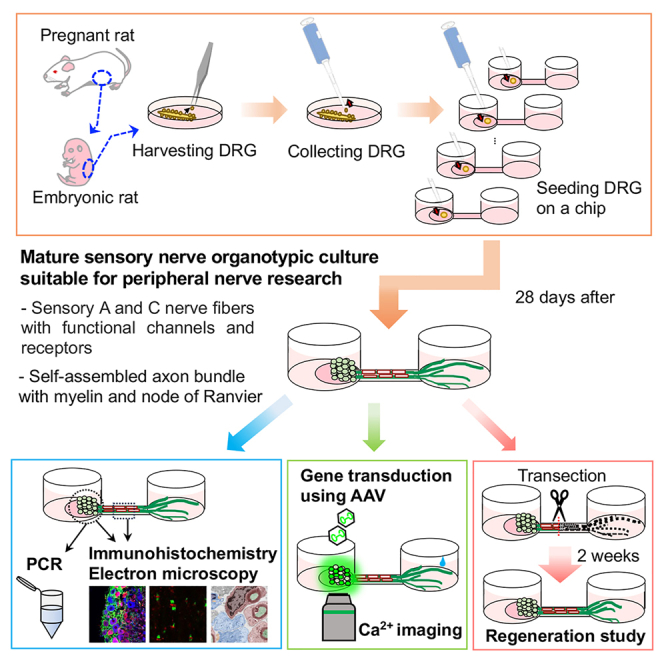

## Teaser

Koyanagi et al. develop a rat dorsal root ganglion (DRG)-derived sensory nerve organotypic model by culturing DRG explants on an organoid culture device. The model recapitulates several structural and functional properties of the peripheral nervous system and can potentially advance peripheral nerve research.

## Introduction

Current gaps in knowledge regarding peripheral neuropathy are in part due to a lack of optimized research tools to study the peripheral nervous system (PNS) that better recapitulate the *in vivo* environment and allow sequential assessment of disease progression. Multiple culture systems are used to study the mechanisms of peripheral neuropathy, including mixed cultures of Schwann cells and dorsal root ganglion (DRG) neurons, DRG explant cultures, and human induced pluripotent stem cell (hiPSC)-derived sensory nerve organoids.^1^^,^^2^^,^^3^^,^^4^ Co-culture of Schwann cells and DRG neurons and DRG explant culture are suitable for *in vitro* analysis of myelination and demyelination, as these platforms develop myelin segments on culture plates.^1^^,^^3^^,^^4^ However, in mixed cultures of Schwann cells and DRG neurons, cells are mixed and scattered on chamber surfaces, precluding analysis of functional changes specific to Schwann cells and neurons. Although conventional DRG explant cultures independently develop ganglion-like structures and nerve axons, the axons extend radially and do not form axon bundles as the peripheral nerves do.^5^^,^^6^ Several recent studies developed *ex vivo* organotypic models derived from rat DRGs using microchip devices and showed the existence of a some myelin in these models.^7^^,^^8^ However, to the best of our knowledge, no previous studies using microfluidics technology have addressed the composition of PNS-specific A fibers and C fibers in organotypic models. Nerve organoids derived from hiPSCs have structural features similar to peripheral nerves, such as independent neuronal cell bodies and axonal bundles.^9^^,^^10^ However, because it is difficult for hiPSC-derived peripheral nerve organoids to stably form myelinating Schwann cells, it remains challenging to use them to study peripheral neuropathy accompanied by demyelination.

Peripheral neuropathy has multiple underlying causes, including diabetes, chemotherapy, and viral infection.^11^^,^^12^^,^^13^ Peripheral neuropathy-associated chronic pain is not simply a persistent state of acute pain but, rather, is induced by complex neuroplastic changes in the PNS, a heterogeneous structure consisting of multiple nerve fiber types (i.e., myelinated Aβ and Aδ fibers and unmyelinated C fibers) and myelinated Schwann cells.^11^^,^^14^^,^^15^^,^^16^^,^^17^^,^^18^ Patients experience hypersensitivity, characterized by symptoms such as hyperalgesia, tingling, and spontaneous pain, in early phases of peripheral neuropathy, which is followed by hypoesthesia and numbness at advanced stages.^12^^,^^17^^,^^19^ However, the mechanisms underlying peripheral neuropathy pathogenesis remain incompletely understood.

To overcome these issues, we developed a rat DRG-derived *ex vivo* sensory nerve organotypic model by combining existing DRG explant culture techniques with a culture device chip that promotes tightly associated axon bundle formation. This methodology enables us to simultaneously generate a dozen to hundreds of sensory organotypic cultures simply by seeding DRG explants derived from rat embryos. The characteristic feature of our *ex vivo* model is that it is comprised of independent ganglion-like structures and self-assembled axonal bundles, including unmyelinated C fibers and stereo-myelinated A fibers, similar to the structural properties of the PNS. We further confirmed the existence of nodes of Ranvier in the model. This organotypic culture allowed evaluation of time-dependent neuroplastic changes specific to the ganglion and nerve axons under more appropriate conditions that are similar to those in the *in vivo* PNS and could be used to investigate the regulatory mechanisms of peripheral neuropathy.

## Results

### Generation of the sensory nerve organotypic model

**Figure 1:**
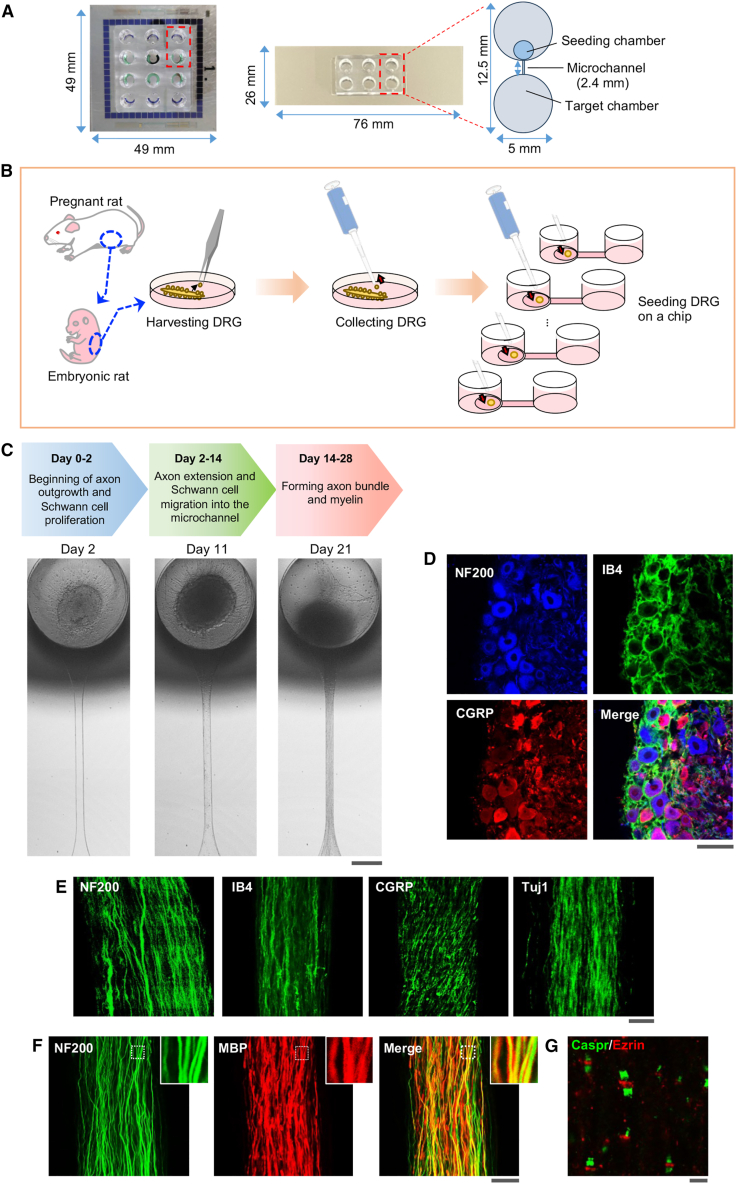
Generation of the sensory nerve organotypic model (A) Photographs of organoid culture microchambers (left, six-well type; center, three-well type) and schematic of the well within the chambers (right). (B) Schematic of the protocol for seeding DRG explants on the organoid culture microchamber. (C) Bright-field images of organotypic model growth at 2, 11, and 21 days following embryonic rat DRG seeding on the culture microchamber. (D–G) Representative confocal images of an organotypic model 28 days after seeding. (D) Images showing NF200-positive (blue), IB4-positive (green), and CGRP-positive (red) neuronal cell bodies in the ganglion-like structure. (E) Images showing Tuj1-positive (green), NF200-positive (green), IB4-positive (green), and CGRP-positive (green) nerve fibers in organotypic axon fascicles. (F) Images showing NF200-positive nerve fibers (green) and MBP-positive myelin (red) in organotypic axon fascicles. (G) Images showing Ezrin puncta (red) at the node of Ranvier and Caspr puncta (green) at paranodes in organotypic axon fascicles. Scale bars: 300 μm (C), 50 μm (D–F), and 25 μm (G).

To obtain a 3-dimensional (3D) sensory nerve organotypic model, we used an organoid culture slide chamber formed on a glass plate (Figure 1A; see STAR Methods for more details). DRG explants derived from embryonic day 15.5 rats were placed in the seeding chambers of each well of the organoid culture device (Figure 1B; see STAR Methods for more details). Embryonic DRG explants contain both sensory neurons and Schwann cells, and we observed elongation of neuronal axons and migration of Schwann cells from DRGs 1–2 days after seeding (Figures 1C and S1A). Neural axons extended spontaneously into the microchannel, accompanied by proliferation and migration of Schwann cells along the neural axons. Axons and Schwann cells reached the other side of the chamber ∼10 days following DRG seeding. At ∼21 days following seeding, thick axon bundles had formed within the microchannels.

Immunostaining revealed that both ganglia and axon bundles were positive for cell-type-specific markers, including nerve filament 200 (NF200; myelinated A fibers), calcitonin gene-related peptide (CGRP; peptidergic C fibers), and isolectin b4 (IB4; non-peptidergic C fibers), which are expressed in DRG neurons *in vivo*^20^^,^^21^^,^^22^ (Figures 1D and 1E). Glial fibrillary acidic protein (a satellite glia cell marker)-positive cells and S100β (a Schwann cell marker)-positive cells also existed within the ganglion-like structure (Figures S1B and S1C). Although CD31-positive blood vessels were not seen in this model, Iba1-positive cells (putative resident macrophages) were present (Figures S1D and S1E). Axon bundles contained abundant neural fibers positive for the pan-neuronal marker tubulin β3 (Tuj1; Figure 1E). In addition, organotypic cultures stained positive for myelin basic protein (MBP), a primary myelin component and myelinating Schwann cell marker,^23^ which co-localized with NF200 in axon bundles (Figure 1F). Nodes of Ranvier are essential structures for saltatory conduction.^24^ In the PNS, ezrin is localized to Schwann cell microvilli on the nodes of Ranvier, and Caspr is localized to the nerve axons at the paranodal junction.^25^^,^^26^ Ezrin puncta were present between separate Caspr puncta (Figure 1G).

### Evaluation of structural properties and gene expression of functional proteins in *ex vivo* sensory organotypic cultures

**Figure 2:**
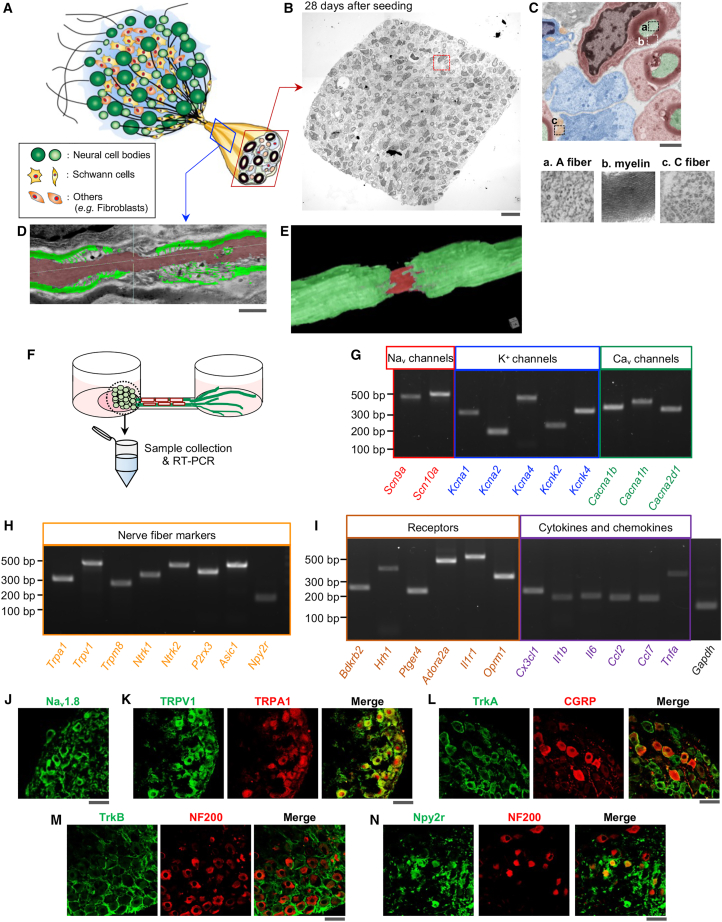
Structural characteristics and ganglion expression of representative ion channels, receptors, and sensory neuron markers in the sensory nerve organotypic model (A) Schematic of the organotypic model, showing its structure. (B) Electron micrographs of an axon bundle cross-section containing abundant myelinated nerve fibers 28 days after seeding. (C) Enlarged images of whole-axon-bundle coronal section (dotted red square in B). A fibers (green), myelinating Schwann cells (red), C fibers (orange) and non-myelinating Schwann cells (blue) were present in the axon bundle. The enlarged views of the A fiber (a), multi-layered myelin sheath (b), and C fiber (c) correspond to the dotted squares in (B). (D) Electron micrographs of an axon bundle sagittal section showing a node of Ranvier 28 days after seeding. (E) 3D reconstructed image of the axon bundle sagittal section in (D), showing a node of Ranvier. (F) Schematic showing sample collection of a ganglion-like structure of an organotypic culture for RT-PCR. (G–I) RT-PCR bands showing mRNA levels of (G) cation channels, (H) nerve fiber markers, (I) functional receptors and pro-nociceptive cytokines and chemokines in ganglia of organotypic cultures 28 days after seeding. GAPDH was used as an internal control. *n = 3*. (J–N) Confocal images showing Nav1.8-positive (J, green), TRPV1-positive (K, green), TRPA1-positive (K, red), TrkA-positive (L, green), CGRP-positive (L, red), TrkB-positive (M, green), NF200-positive (M and N, red), and Npy2r-positive (N, green) neuronal cell bodies in the ganglion-like structure of an organotypic model 28 days after seeding. Scale bars: 10 μm (B), 500 nm (C), 1 μm (D), and 50 μm (J–N).

Electron microscopy revealed numerous myelinated nerve fibers in cross-sections of the organotypic axon bundles (Figures 2A–2C). Within the axon, scattered neurofilaments or microtubules,^27^ which appeared as hollow circles, were detected in cross-sections (Figure 2C). Multi-layered myelin sheets surrounded medium- to large-diameter nerve fibers such as A fibers, and Remak Schwann cells^28^^,^^29^ were adjacent to small-diameter fibers such as C fibers (Figures 2B and 2C). Nodes of Ranvier^30^^,^^31^ were also present in sagittal sections of the axon fascicles (Figure 2D). 3D reconstructed images revealed nodal gaps between myelin sections in axon bundles of the organotypic culture (Figure 2E).

To determine whether the sensory nerve organotypic cultures express ion channels, receptors, cytokines, and chemokines essential for sensory nerve function, the ganglion-like structure of the organotypic model was physically separated and collected 28 days after seeding and then subjected to PCR analysis (Figure 2F). The ganglia expressed voltage-gated sodium (Na_v_1.7/*Scn9a* and Na_v_1.8/*Scn10a*) and potassium channels (K_v_1.1/*Kcna1*, K_v_1.2/*Kcna2*, K_v_1.4/*Kcna4*, TREK-1/*Kcnk2,* and TRAAK1/*Kcnk4*) (Figure 2G), which are involved in initiation and rapid propagation of action potentials.^32^^,^^33^^,^^34^ The organotypic culture also expressed N-type calcium channels (Ca_v_2.2/*Cacna1b*) and T-type calcium channels (Ca_v_3.2/*Cacna1h*), which are related to pain signal transmission, and α2δ subunits (*Cacna2d1*), the therapeutic target of gabapentin (Figure 2G).^35^^,^^36^ The organotypic culture expressed representative C fiber markers such as transient receptor potential (TRP) channels (*Trpa1*, *Trpv1*, and *Trpm8*)^37^ and purinergic receptor P2X3 (*P2rx3*)^38^ and the A fiber marker neuropeptide Y receptor type 2 (*Npy2r*),^39^ in addition to high-affinity neurotrophin receptors (TrkA/*Ntrk1* and TrkB/*Ntrk2*)^14^ and acid-sensing ion channel subunit 1 (*Asic1*)^14^^,^^40^ (Figure 2H). Further, we detected mRNA expression of receptors related to inflammation (bradykinin receptor B2 [*Bdkrb2*], histamine receptor H1 [*Hrh1*], prostaglandin E receptor 4 [*Ptger4*], adenosine A2a receptor [*Adora2a*], and interleukin-1 receptor type 1 [*Il1r1*])^41^^,^^42^^,^^43^^,^^44^^,^^45^ and *Oprm1*, which encodes the μ-opioid receptor^46^ (Figure 2I). Additionally, mRNA expression of pro-inflammatory cytokines and chemokines (*Cx3cl1*, *Il1b*, *Il6*, *Ccl2*, *Ccl7*, and *Tnfa*), which are involved in pain signaling,^44^^,^^47^^,^^48^^,^^49^^,^^50^^,^^51^ was detected in the organotypic cultures (Figure 2I). Immunohistochemical data further confirmed that Nav1.8, TRPA1, TRPV1, TrkA, TrkB, and Npy2r were expressed in the ganglion-like structure of the organotypic model, similar to the expression patterns of these markers in rodent DRGs^39^^,^^52^^,^^53^^,^^54^ (Figures 2J–2N). TRPV1 and TRPA1 were detected in almost the same population. TrkA, which is expressed in peptidergic C fibers and nociceptive A fibers,^55^ co-localized with CGRP-positive neurons. TrkB and Npy2r were expressed on the cell surface and in the cytosol of NF200-positive A fibers, respectively. The mRNA level of Hopx, a transcription factor that is highly expressed in differentiated sensory neurons,^56^ was higher in mature organotypic cultures than in embryonic day 15.5 (E15.5) rat DRGs (Figure S2).

### Evaluation of neuronal excitation in organotypic neural cell bodies after topical chemical application onto nerve endings

**Figure 3:**
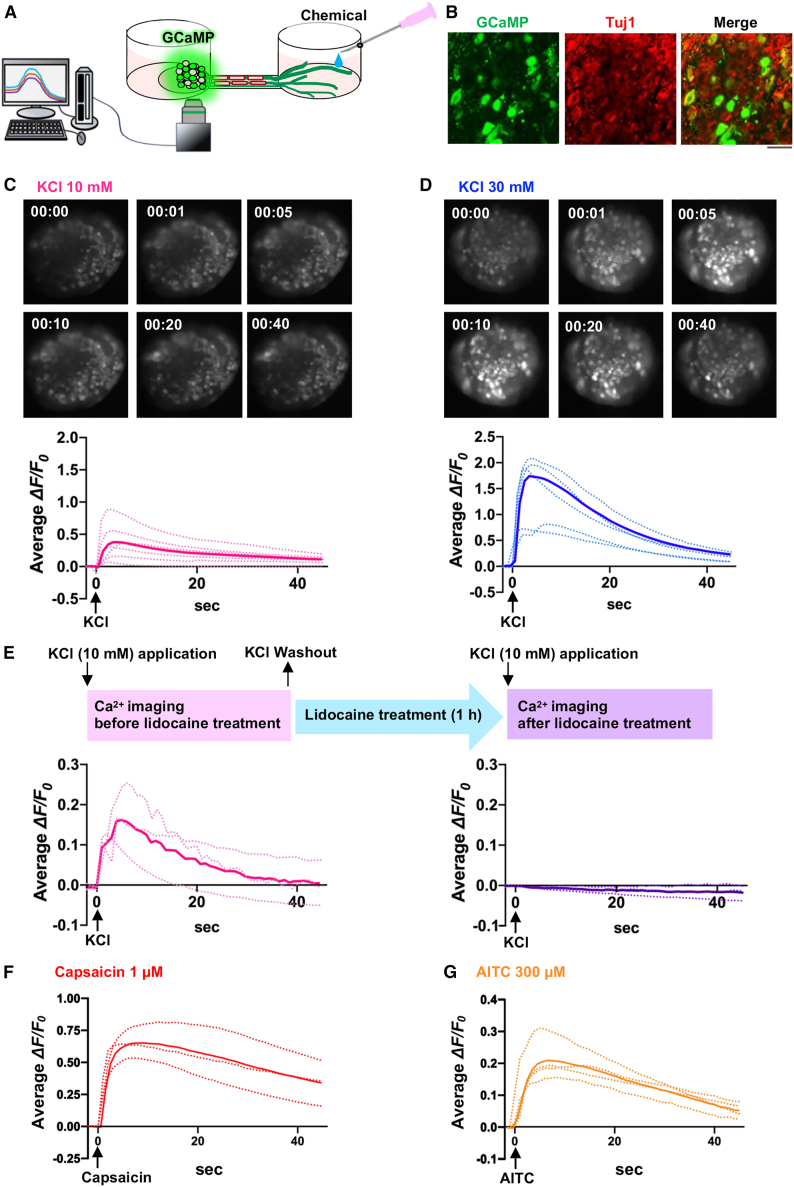
Chemical stimulus-induced neuronal excitation (A) Schematic showing measurement of GCaMP6m fluorescence in neuronal cell bodies within the ganglion-like structure. (B) Confocal images showing GCaMP6m (green)- and Tuj1 (red)-positive neuronal cell bodies in the ganglion-like structure of an organotypic culture after AAV-hSyn-GCaMP6m infection. See also Figure S3. Scale bar: 50 μm. (C and D) GCaMP6m fluorescence micrographs (top) and traces (bottom) indicating Ca^2+^ responses in neuronal cell bodies of organotypic cultures upon KCl treatment (C, 10 mM; D, 30 mM). *n = 6* (C) and 5 (D). (E) GCaMP6m fluorescence traces showing Ca^2+^ responses in organotypic neuronal cell bodies upon KCl application (10 mM) before and after lidocaine treatment (3 mM). *n = 3*. (F and G) GCaMP6m fluorescence traces showing Ca^2+^ responses in neuronal cell bodies of organotypic cultures upon capsaicin (1 μM, F) and AITC (300 μM, G). *n = 3* (F) and 4 (G). Dotted and bold traces show data from individual organotypic cultures and the mean of six (C), five (D), three (E and F), and four (G) organotypic cultures.

Subsequently, we conducted Ca^2+^ imaging analysis to evaluate changes in Ca^2+^ influx into organotypic neural cell bodies upon chemical stimulation of nerve endings (Figure 3A). KCl was used as an activator to depolarize both myelinated A fibers and non-myelinated C fibers. Organotypic neural cells were selectively transduced with an adeno-associated virus (AAV) encoding a genetically encoded calcium indicator, GCaMP6m, under the neuron-specific synapsin promoter (AAV-hSyn-GCaMP6m; Figure S3).^57^ GCaMP6m fluorescence was observed within Tuj1-positive neural cell bodies in the organotypic model (Figure 3B). Topical KCl application (10 and 30 mM) induced a rapid increase of GCaMP6m fluorescence in both large-diameter and small-diameter cells, indicating a [Ca_2+_]_i_ increase in neuronal cell bodies of the model (Figures 3C and 3D). Organotypic cultures exhibited higher *ΔF/F*_*0*_ following treatment with 30 mM KCl than with 10 mM KCl, suggesting a concentration-dependent organotypic response to the stimuli. Further, these responses were abolished by pre-treatment with lidocaine (3 mM), an amide local anesthetic (Figure 3E). Transient increases of GCaMP6m fluorescence were also elicited by local application of the TRPV1 agonist capsaicin and the TRPA1 agonist allyl isothiocyanate (AITC) (Figures 3F and 3G).

### Time course evaluation of axon bundle regeneration and remyelination following axotomy

**Figure 4:**
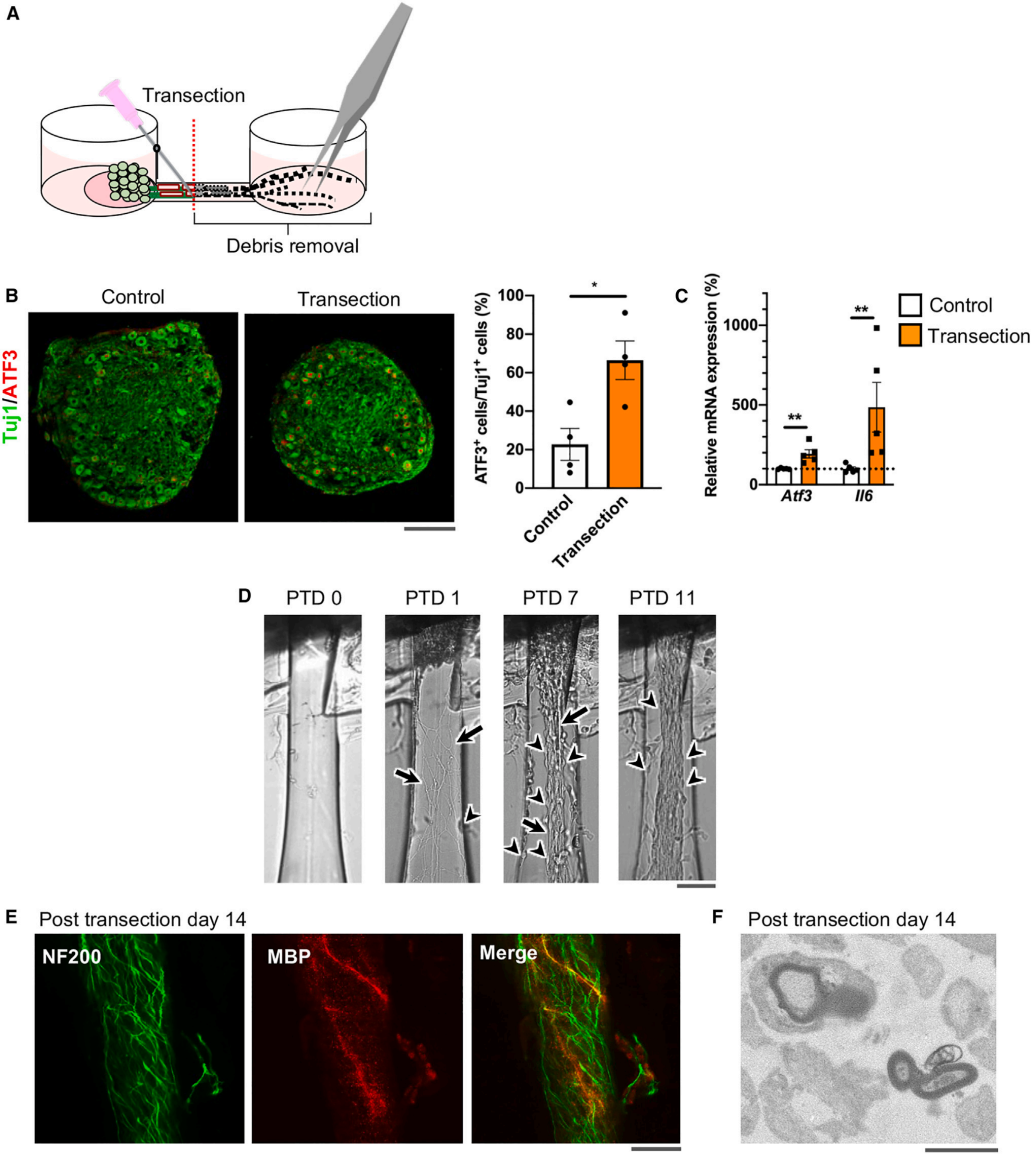
Gene expression changes and regeneration of axon bundle nerve fibers and myelin after transection (A) Changes in ganglion-like structure gene expression were analyzed after axon bundle transection in the middle of the microchannel. (B) Fluorescence micrographs of Tuj1 (green) and ATF3 (red) immunoreactivity (left) and percentage of ATF3-positive neurons among Tuj1-positive neurons (right) in ganglion-like structures of organotypic cultures 1 day following axon bundle transection. Data are expressed as means ± SEM. ∗*p* < 0.05 vs. control (non-transected organoid). *n = 4*. (C) Quantitative real-time PCR analysis of *Atf3* and *Il6* mRNA levels in ganglion-like structures of organotypic cultures 1 day after axonal transection. Data are expressed as means ± SEM. ∗∗*p* < 0.01 vs. control. *n = 5*. (D) Bright-field images showing axon bundle regeneration 0, 1, 7, and 11 days after transection. Regenerating axons and migrating Schwann cells are indicated by black arrows and arrowheads, respectively. PTD, post-transection day. (E) Confocal images showing NF200 (green) and MBP (red) immunoreactivity in a regenerated axon bundle 14 days after axonal transection. (F) Electron micrographs of a regenerated axon bundle cross-section showing regeneration of stereo-myelin 14 days after axonal transection. Scale bars: 200 μm (B), 100 μm (D), 50 μm (E), and 10 μm (F).

The organotypic cultures were subjected to axonal transection and debris removal 28 days after seeding (Figure 4A). Immunoreactivity of activating transcription factor 3 (ATF3), a stress-induced transcriptional factor,^58^^,^^59^ increased in ganglion neuronal cells 1 day following axonal transection (Figure 4B). Expression of *Atf3* mRNA was higher in ganglia of the transected organotypic model than in ganglia of the mature organotypic model without transection and the E15.5 rat DRG explant (Figures 4C, and S4A). Axonal transection also increased mRNA expression of interleukin-6, a pro-inflammatory cytokine that is upregulated in the DRGs following nerve injury *in vivo*^47^^,^^60^ (Figure 4C). Axon regeneration, indicated by a linear morphology, and Schwann cell migration, indicated by a circular morphology, began 1 day after axonal transection (Figure 4D). These migrating Schwann cells were immunopositive for the Schwann cell marker S100β^61^^,^^62^ (Figure S4B). The regenerating axons and migrating Schwann cells increased gradually and formed a regenerated axon bundle over 14 days following axonal transection. In the regenerated axon fascicles, myelin formation and NF200-positive sensory A fibers were detected by confocal microscopy (Figure 4E). Electron microscopy also clearly demonstrated the presence of multi-layered myelin sheaths in the axon bundle 14 days following transection (Figure 4F).

## Discussion

The main feature of the production method of our organotypic culture system is that a large number of organoids can be produced simultaneously with high reproducibility simply by seeding DRG explants derived from rat embryos on a microchamber. The present findings indicate that this sensory organotypic culture could be used for analysis of physiological responses to pain-related stimuli, such as changes in gene expression and neural activity, or for continuous assessment of morphological changes related to pain development and nerve regeneration.

Previously, multiple sensory nerve organoids and organotypic models have been used to investigate peripheral neuropathy. Recent studies have established a model in which hiPSC-derived sensory neurons and rat Schwann cells are co-cultured to form myelin sheaths around unidirectionally elongated nerve axons.^2^ Additionally, prior studies have generated sensory organotypic models by inducing myelination around nerve axons in 3D hydrogel microfluidics culture of rat DRG explants.^7^^,^^8^ hiPSC-derived organoids would have the advantages that they can retain human tissue characteristics and replicate the pathology of diseases in a patient-specific manner.^63^^,^^64^ Although these models self-assemble cell bodies and axons, myelinated A fibers, unmyelinated C fibers, and myelin sheaths that form nodes of Ranvier, which are essential structures of the PNS, are not detected. Recently, a hiPSC-derived sensory nerve organoid was developed using a microfluidics device; however, it does not contain myelin.^10^ Another study that developed an iPSC-derived assembloid containing sensory neurons and associated Schwann cells did not show the existence of myelin using a myelin-specific marker.^65^ Importantly, in contrast with the other models, our *ex vivo* culture has the major structural feature of *in vivo* sensory nerves. Myelinated A fibers, non-myelinated C fibers in the ganglia, and axon fascicles were present in the organotypic model. The organotypic model also contained satellite glia cells, Schwann cells, and residential macrophages, although blood vessels were not observed. Furthermore, Schwann cells formed stable multi-layered stereo-myelin around A fibers, which was observed throughout the cross-sections of organotypic axon bundles. Unlike in other models, these 3D myelin sheaths formed nodes of Ranvier, which enable saltatory conduction along nerve axons in the PNS. The insight that the organotypic models exhibited many similarities to the basic properties of peripheral nerves *in vivo* was further supported by the immunoreactivity and abundant mRNA levels of representative ion channels, receptors, cytokines, and chemokines, which are important for the maintenance of sensory nerve function.^14^^,^^32^^,^^33^^,^^34^^,^^35^^,^^36^^,^^37^^,^^38^^,^^39^^,^^40^^,^^41^^,^^42^^,^^43^^,^^44^^,^^45^^,^^46^^,^^47^^,^^48^^,^^49^^,^^50^^,^^51^^,^^60^ To elucidate the complex mechanism of polyneuropathy with pain and hypoesthesia symptoms, we think it is necessary to use a culture system containing multiple sensory nerve fiber types and stereo-myelin. Individual fluorescent labeling of each nerve fiber and Schwann cell in organotypic cultures and time-lapse analysis of their pathological changes will greatly help us to understand the pathogenesis of peripheral neuropathy. The organotypic culture developed in this study has great advantages for promoting such research.

In the present study, we introduced genes specifically into neural cells of the organotypic culture using AAV-hSyn-GCaMP6m.^57^ This AAV-based gene transformation enabled time-lapse analysis of changes in neuronal activity upon depolarizing stimuli to nerve endings based on changes in ganglion GCaMP fluorescence intensity. Furthermore, we demonstrated that axonal transection induced expression of pain-related molecules, similar to changes in the DRGs following nerve injury *in vivo*.^47^^,^^58^^,^^59^ These compelling findings suggest afferent transduction of pain-related signals from nerve terminals to cell bodies in the organotypic model. Further, KCl-induced Ca^2+^ influx into the organotypic cell bodies was almost completely abolished by lidocaine pre-treatment. Thus, the organotypic model has potential utility for screening of analgesic drug candidates based on the transduction of pain-related responses in the cell body in response to stimulation and the efficacy of a proof-of-concept anesthetic. Together with immunohistochemical data indicating expression of functional channels and receptors, the data showing that TRPV1 and TRPA1 agonists induce Ca^2+^ signaling in cell bodies further emphasize that this organotypic model retains the functional characteristics of peripheral nerves.

Myelinating Schwann cells are capable of transformation into an immature state in response to peripheral nerve injury.^66^^,^^67^ Schwann cells play a crucial role in the outgrowth and guidance of regrowing peripheral axons following injury.^67^^,^^68^ We demonstrated that, in the organotypic cultures, injured nerve axons began to elongate within the microchannel accompanied by the migration of Schwann cells 1 day after transection and formed axon bundles within 14 days. Confocal and electron microscopic analyses revealed that multi-layered myelin formed around nerve fibers in regenerated axon bundles 14 days following transection, indicating that the organotypic model possessed regeneration capacities similar to the PNS. These data highlight the utility of this organotypic culture for long-term analysis of morphological changes related to nerve impairment and regeneration.

As indicated by expression of *Hopx* and *Atf3* mRNA, the mature organotypic DRG explant had different molecular characteristics than E15.5 rat DRG explants, which are in a relatively immature state with myelination/maturation of Schwann cells not yet completed.^69^ In particular, *Atf3* mRNA was detected in cell bodies in the organotypic culture after axonal transection but not in E15.5 rat DRG explants. Thus, it is presumed that the axonal transection process in the mature organotypic model is different from the dissection and isolation process of E15.5 DRGs and that the latter process does not result in excessive cumulative damage of the DRG explant. In addition, our results showing that the organotypic model contains residential-like macrophages raise the possibility that this culture system is useful for investigating the PNS-immune system interaction associated with the development of neuropathic pain and nerve regeneration.

As demonstrated in recent studies, there are both similarities and differences in expression patterns of sensory nerve markers between humans and rodents.^70^^,^^71^^,^^72^ When comparing mouse and human, differences in the distribution of sensory nerve subpopulations (e.g., TrkA-, CGRP-, or P2X3R-positive neurons) have been reported.^71^^,^^72^ On the other hand, it has been shown that 80% of proteins are shared across species between rat and human DRG.^70^ Based on these facts, although we need to consider the species differences between humans and rodents, the organotypic model developed in this study secures its advantages in peripheral nerve research. It would be possible to apply the techniques shown in this study to non-human primate DRGs and to develop *ex vivo* culture closer to the human DRG.

In conclusion, our findings demonstrated that the sensory organotypic model developed in this study showed some structural and functional similarities to peripheral sensory nerves. This platform not only enabled analysis of changes in Ca^2+^ signaling in response to noxious stimuli but also time-dependent morphological changes of nerve fibers and myelin under pathological conditions simulating peripheral neuropathy pathogenesis. Thus, this organotypic model will yield crucial insights into the complex mechanisms underlying peripheral neuropathy at the early and refractory phases and mechanistic findings could lay the groundwork for more targeted approaches to treatment.

### Limitations of the study

The organotypic model has limitations that should be addressed further. First, DRG neurons have been known to be pseudounipolar, with axonal branches extending into the peripheral tissue and spinal cord. We cannot determine whether our *ex vivo* cultures have both peripheral and central axonal branches extending into the microchannels of the chip or are simply unipolar with one axonal bundle. Recently, Rockel et al. have developed an iPSC-derived assembloid that is composed of mesenchymal tissue and peripheral tissue containing pseudounipolar sensory neurons.^65^ Considering their findings, it would be possible to address the issue in our future study. Second, we confirmed mRNA expression of various functional molecules in the organotypic model, but the cellular distributions of ion channels and receptors on nerve fibers and secretion of cytokines and chemokines were not fully investigated. Third, to evaluate neuronal excitability in response to noxious axon stimulation, electrophysiological approaches to analyze nerve conduction velocity are required in addition to Ca^2+^ imaging analysis. We are presently developing a specialized organoid/organotypic culture chamber to allow it.

## STAR★Methods

### Key resources table

**Table:** 

REAGENT or RESOURCE	SOURCE	IDENTIFIER
**Antibodies**		

Mouse anti-NF200	Sigma-Aldrich	Cat#N0142; RRID: AB_477257
Rabbit anti-CGRP	Sigma-Aldrich	Cat #C8198; RRID: AB_259091
Isolectin B4 (Ib4)-FITC conjugate	Sigma-Aldrich	Cat#L2895; RRID: AB_2314664
Rabbit anti-MBP	Abcam	Cat #ab40390; RRID: AB_1141521
Mouse anti-Caspr, clone K65/35	Merck	Cat #MABN69; RRID: AB_10806491
Rabbit anti-Ezrin	Cell Signaling Technology	Cat #3145; RRID: AB_2100309
Mouse anti-Tubulin β3	BioLegend	Cat #801201; RRID: AB_2313773
Rabbit anti-Nav1.8	Alomone labs	Cat#ASC-016-GP; RRID: AB_2040188
Guinea pig ant-TRPV1	Neuromics	Cat #GP14100; RRID: AB_1624142
Rabbit anti-TRPA1	Novus Biologicals	Cat#NB110-40763; RRID: AB_715124
Goat anti-Trka	R&D systems	Cat#AF1056; RRID: AB_2283049
Goat anti-Trkb	R&D systems	Cat#AF1494; RRID: AB_2155264
Rabbit anti-Npy2r	Sigma-Aldrich	Cat#SAB4502029; RRID: AB_10747296
Rabbit anti-GFAP	Abcam	Cat#ab7260; RRID: AB_305808
Rabbit anti-S100β	Abcam	Cat#ab52642; RRID: AB_882426
Rabbit anti-Iba1	Fujifilm Wako Pure Chemical Industries	Cat#019–19741; RRID: AB_839504
Mouse anti-CD31	Cell Signaling Technology	Cat#3528; RRID: AB_2160882
Rabbit anti-ATF3	Sigma-Aldrich	Cat #HPA001562; RRID: AB_1078233
Alexa Fluor 488 goat anti-mouse	Thermo Fisher Scientific	Cat#A28175; RRID: AB_2536161
Alexa Fluor 488 goat anti-rabbit	Thermo Fisher Scientific	Cat#A-11008; RRID: AB_143165
Alexa Fluor 488 donkey anti-goat	Thermo Fisher Scientific	Cat#A28175; RRID: AB_2534102
Alexa Fluor 594 goat anti-mouse	Thermo Fisher Scientific	Cat#A-11032; RRID: AB_2534091
Alexa Fluor 594 goat anti-rabbit	Thermo Fisher Scientific	Cat#A-11012; RRID: AB_2534079
Alexa Fluor 405 goat anti-mouse	Thermo Fisher Scientific	Cat#A-31553; RRID: AB_221604

**Bacterial and virus strains**		

AAV-hSyn-GCaMP6m	Addgene viral prep	Cat#100841-AAV9

**Chemicals, peptides, and recombinant proteins**		

KCl	Nacalai Tesque	Cat#28513-85
Lidocaine	Fujifilm Wako Pure Chemical Industries	Cat#120-02691
Capsaicin	Sigma-Aldrich	Cat#M2028
Allyl isothiocyanate	Fujifilm Wako Pure Chemical Industries	Cat#016-01463
Poly-*l*-lysin hydrobromide	Sigma-Aldrich	Cat#P1524
Natural Mouse Laminin	Thermo Fisher Scientific	Cat#23017015
DMEM	Fujifilm Wako Pure Chemical Industries	Cat#044-29765
Penicillin–streptomycin	Nacalai Tesque	Cat#09367-34
MACS neuro medium	Miltenyi Biotec	Cat#130-093-570
MACS NeuroBrew-21	Miltenyi Biotec	Cat#130-093-566
GlutaMAX	Thermo Fisher Scientific	Cat#35050061
NGF-2.5S from murine submaxillary gland	Sigma-Aldrich	Cat#N6009
Ascorbic acid	Sigma-Aldrich	Cat#A5960
Forskolin	Sigma-Aldrich	Cat#F6886
PFA	Nacalai Tesque	Cat#26126-54
Methanol	Nacalai Tesque	Cat#21915-93
BSA	Nacalai Tesque	Cat#01281-97
Glutaraldehyde	Nacalai Tesque	Cat#17025-25
Osmium tetroxide	Nacalai Tesque	Cat#25746-06
Uranyl acetate	Merck	Cat#8473
Epoxy resin(Luveak-812)	Nacalai Tesque	Cat#20829-05
Ethanol	Nacalai Tesque	Cat#14712-63
Lead(Ⅱ) Nitrate	Nacalai Tesque	Cat#20231-02
Sodium citrate, tribasic	Nacalai Tesque	Cat#314-04

**Deposited data**		

Raw data for Figures in the main manuscript	This paper	Mendeley Data: https://doi.org/10.17632/gcksz464sh.1

**Experimental models: Organisms/strains**		

Slc:Wistar/ST rats	Japan SLC	RS:0002216

**Oligonucleotides**		

Primers for RT-PCR	This paper	Tables S1–S3

**Software and algorithms**		

HCImage	Hamamatsu Photonics	https://hcimage.com
NIS-Elements	Nikon	https://www.microscope.healthcare.nikon.com/products/software/nis-elements
Dragonfly	Oneida Research Services, Inc.	http://www.theobjects.com/dragonfly/
GraphPad Prism 8	GraphPad Software	https://www.graphpad.com/

**Other**		

Organoid-culturing device	SHARP Co.	Cat#LF0DAS0227

### Resource availability

#### Lead contact

Further information and requests for resources and reagents should be directed to and will be fulfilled by the Lead Contact, Satoshi Imai (imais06@wakayama-med.ac.jp).

#### Materials availability

This study did not generate unique reagents.

#### Data and code availability

• Raw data for the Figures in the main manuscript has been deposited at Mendeley Data and is publicly available. The DOI is: https://doi.org/10.17632/gcksz464sh.1. All data reported in this paper will be shared by the lead contact upon request.
• This paper does not report original code.
• Any additional information required to reanalyze the data reported in this paper is available from the lead contact upon request.

### Experimental model and study participant details

#### Animals

All animal experiments were approved by the Kyoto University Animal Research Committee (permission number: 23–75) or Wakayama Medical University Animal Care and Use Committee (permission number: TORA-53) and performed according to the guidelines of the animal ethics committee of Kyoto University and Wakayama Medical University, respectively. Pregnant Wistar/ST rats were purchased from Japan SLC (Shizuoka, Japan). All animals were housed under a 12 h light–dark cycle at a constant ambient temperature (24°C ± 1°C) and humidity (55% ± 10%), and allowed access to food and water *ad libitum*. All reasonable efforts were made to minimize the number of animals used and to limit experimentation to necessary studies.

#### AAV

AAV.Syn.GCaMP6m.WPRE.SV40 (AAV-hSyn-GCaMP6m) was a gift from Douglas Kim & GENIE Project (Addgene viral prep #100841-AAV9; http://n2t.net/addgene:100841; RRID:Addgene_100841).

### Method details

#### Drugs and chemicals

KCl (Nacalai Tesque, Kyoto, Japan) was dissolved in distilled water as a stock solution at a concentration of 1 M. Lidocaine, an amide local anesthetic, (Fujifilm Wako Pure Chemical Industries, Osaka, Japan), and capsaicin were dissolved in DMSO (Nacalai Tesque) as a stock solution at a concentration of 100 mM, and 1 mM, respectively. Each chemical solution was diluted in the appropriate culture medium to achieve the desired concentration before use.

#### Formation of the sensory nerve organotypic model

The organoid-culturing device (SHARP Co., Osaka, Japan, Cat#LF0DAS0227 which is available via Nacalai Tesque; https://www.e-nacalai.jp/ec2/EC-srchAll.cfm?srchword=LF0DAS0227&Kensu=20&Web=J) was used to induce the formation of the *ex vivo* sensory nerve organotypic cultures. This culture slide chamber is applicable for both an iPSC-derived nerve organoid and an organotypic neuronal culture derived from an embryonic tissue. The device is composed of chambers and microchannels formed by poly(dimethylsiloxane) (PDMS) on alkali-free glass slides. Each well consisted of two circular chambers: a seeding chamber to place a spheroid or tissue explant, and a target chamber to accommodate axon terminals, connected by a 2.4-mm microchannel for axon fascicle formation. Each chamber can maximally retain 200 μL of media in it. The cultures were maintained by 150 μL of media (i.e., approximately 75 μL of media in each chamber) in this study. Before starting culture, the devices were sterilized by washing with 70% ethanol and UV light irradiation for at least 1 h. Then, the devices were coated with 150 μL of 0.1 mg/mL poly-*l*-lysin (Sigma-Aldrich, St. Louis, MO, USA) and 12.5 μg/mL laminin (Thermo Fisher Scientific, Waltham, MA, USA) at 37°C and 5% CO_2_ for 12 h∼ and 4 h∼, respectively. Poly-*l*-lysin and laminin were removed and then washed three times with ∼150 μL of PBS.

Pregnant rats were deeply anesthetized with isoflurane and uteri were collected. Embryonic day 15 Wistar/ST rat pups were obtained from uteri and transferred into 100 mm dish containing DMEM (Fujifilm Wako Pure Chemical Industries) supplemented with 10% FBS (Thermo Fisher Scientific) and 1% penicillin–streptomycin (P/S; Nacalai Tesque) on ice. Under a microscope, a ventral side of backbone was opened from rostral end. Spinal cord with DRG was removed from the backbone and pooled in the DMEM supplemented with 10% FBS and 1% P/S on ice. Lumbar DRG were harvested from spinal cord just before seeding. A harvested DRG was collected using a micropipette adjusted as 10 μL and plated onto each seeding chamber of the organoid-culturing device.

According to the previous report,^4^ the following culture media were used with slight modifications (See Figure S1A). The devices were filled with 150 μL of culture media containing MACS neuro medium (Miltenyi Biotec, Bergisch Gladbach, Germany) supplemented with 10% FBS (Thermo Fisher Scientific), 1% penicillin–streptomycin (P/S; Nacalai Tesque), and 100 ng/mL 2.5 S nerve growth factor (NGF; Sigma-Aldrich) before seeding DRG. After 2 days of culture, the culture media were replaced with 150 μL of ‘neural culture media’ composed of MACS neuro medium containing 2% MACS NeuroBrew-21 (Miltenyi Biotec), 0.5% GlutaMAX (Thermo Fisher Scientific), 1% P/S, and 100 ng/mL 2.5 S NGF. The media were switched to 150 μL of the neural culture media supplemented with 10% FBS and 0.5 μM forskolin (Sigma-Aldrich) 6 days after seeding DRG. The media were supplemented with 50 μg/mL ascorbic acid (Sigma-Aldrich) to induce myelination from 14 days after seeding.^1^^,^^3^ Throughout the culturing period, each culture medium was replaced to the fresh one every 2–3 days. The bright field images of the organotypic model shown in Figure 1C were obtained using an all-in-one microscope BZ-9000 (KEYENCE, Osaka, Japan). In this research, the organotypic cultures were used for the following experiments at least 28 days after DRG seeding on the device.

#### Immunohistochemistry

The organotypic model was collected from the culture devices using microforceps. After collecting, organotypic model was transferred into a 24 well plate and fixed in 4% paraformaldehyde (PFA) in 0.1 M phosphate buffer (PB) for 20 min to stain axon fascicles of the model. To stain caspr and ezrin, the organotypic cultures were fixed in 100% methanol for 10 min at −20°C as previously described.^73^

For staining of the ganglion-like structure of organotypic model, it was fixed in 4% PFA for 20 min (for staining for ATF3 and Tuj1) or overnight (for staining for NF200, CGRP, Ib4, Nav1.8, TRPV1, TRPA1, TrkA, TrkB, Npy2r, GFAP, and S100β), and permeated with 15% sucrose solution in 0.1 M PB for 3h at 4°C. The specimen was frozen in an embedding compound (Sakura Fintek, Inc., Torrance, CA, USA) after fixation, and cut using a freezing cryostat (Leica CM 1850; Leica Microsystems Inc., Wetzlar, Germany; 16 μm thick) and thaw-mounted on MAS-coated glass slides (Matsunami Glass Ind., Osaka, Japan).

To stain S100β-positive Schwann cells in the regenerating organotypic culture after axonal transection, the culture was fixed in the culturing chamber in 4% PFA for 30 min.

The samples were blocked in blocking buffer (PBS containing 0.1% Tween 20 and 3% BSA) for 1 h at room temperature, and then incubated for 24 h at room temperature with the following primary antibodies: mouse anti-NF200 (1:400, Sigma-Aldrich, #N0142, RRID:AB_477257), rabbit anti-CGRP (1:200, Sigma-Aldrich, #C8198, RRID:AB_259091), Isolectin B4 (Ib4)-FITC conjugate (1:250, Sigma-Aldrich, #L2895), rabbit anti-MBP (1:1000, Abcam, Cambridge, UK, #ab40390, RRID:AB_1141521), mouse anti-Caspr, clone K65/35 (1:100, Merck, Darmstadt, Germany, #MABN69, RRID:AB_10806491), rabbit anti-Ezrin (1:200, Cell Signaling Technology, Danvers, MA, USA, #3145, RRID:AB_2100309), mouse anti-Tubulin β3 (1:500, BioLegend, San Diego, CA, USA, #801201, RRID:AB_2313773), rabbit anti-Nav1.8 (1:200, Alomone labs, Jerusalem, Israel, #ASC-016-GP, RRID:AB_2040188), guinea pig anti-TRPV1 (1:200, Neuromics, Edina, MN, USA, #GP14100, RRID:AB_1624142), rabbit anti-TRPA1 (1:200, Novus Biologicals, Centennial, CO, USA, #NB110-40763, RRID:AB_715124), goat anti-TrkA (1:200, R&D systems, Minneapolis, MN, USA, #AF1056, RRID:AB_2283049), goat anti-TrkB (1:200, R&D systems, #AF1494, RRID:AB_2155264), rabbit anti-Npy2r (1:50, Sigma-Aldrich, #SAB4502029, RRID:AB_10747296), rabbit anti-GFAP (1:1000, Abcam, #ab7260, RRID:AB_305808), rabbit anti-S100β (1:1000, Abcam, #ab52642, RRID:AB_882426), rabbit anti-Iba1 (1:500, Fujifilm Wako Pure Chemical Industries, #019–19741, RRID:AB_839504), mouse anti-CD31 (1:500, Cell Signaling Technology, #3528, RRID:AB_2160882) or rabbit anti-ATF3 (1:200, Sigma-Aldrich, #HPA001562, RRID:AB_1078233). After washing three times with PBS, the samples were incubated for 3 h at room temperature with appropriate secondary antibodies conjugated with Alexa Fluor 594, 488 and/or 405 (1:200, Thermo Fisher Scientific). After washing, samples were mounted in Vectashield (Vector Laboratories, Burlingame, CA, USA), and images were acquired under a laser scanning confocal microscope (A1RMP; Nikon Corporation, Tokyo, Japan).

#### Transmission electron microscopy (TEM)

TEM was performed as previously described.^74^^,^^75^ Briefly, the organotypic cultures were fixed with 4% PFA (Nacalai Tesque) and 2% glutaraldehyde (Nacalai Tesque) in 0.1 M phosphate buffer at 4°C overnight. Specimens were then post-fixed with 1% osmium tetroxide (Nacalai Tesque). After post-fixation, tissues were dehydrated in a graded series of ethanol (50%, 60%, 70%, 80%, 90%, 99%, and 100%), and embedded in epoxy resin (Luveak-812, Nacalai Tesque) according to a standard procedure. Ultra-thin sections were made using an ultramicrotome (UC7, Leica Microsystems Inc.). Sections were stained with uranyl acetate (Nacalai Tesque) and lead citrate, and observed under an H-7650 electron microscope (Hitachi, Ltd., Tokyo, Japan).

#### Scanning electron microscopy array tomography (SEM-AT)

The 90 nm serial sections were collected on a cleaned silicon wafer strip held by a micromanipulator (MN-153, NARISHIGE, Tokyo, Japan). The sections were stained at room temperature using 2% aqueous uranyl acetate for 20 min and Reynolds’ lead citrate for 2 min. The sections were imaged using a SEM (JSM-7900F, JEOL Ltd., Tokyo, Japan) supported by Array Tomography Supporter software (System In Frontier Inc., Tokyo, Japan) that enables automated imaging. For 3D reconstruction, the images were stacked in order by Stacker *NEO* software (System In Frontier Inc.) and the resultant image stacks were processed using Dragonfly Pro software (Oneida Research Services, Inc., Whitesboro, NY, USA).

#### RT-PCR assay

Total RNA was isolated from the ganglion-like structure of organotypic cultures using the SV Total RNA Isolation system (Promega, Madison, WI, USA). The organotypic cultures were collected from the culture devices using microforceps, and then, axon fascicles were removed from ganglion-like structures. To obtain the sufficient amount of RNA, each RNA sample was extracted from ganglion-like structures of 6 organotypic cultures. The following RT-PCR assays were performed as previously described.^3^ Purified 0.2 μg of total RNA was used to prepare first strand cDNA and each target gene was amplified in a 50 μL PCR solution containing 2 mM MgCl_2_, 0.2 mM dNTP mix, and DNA polymerase (Blend Taq, TOYOBO, Japan) along with synthesized primers according to the GenBank sequence as described in Tables S1–S3. GAPDH was used as a normalization control.

#### Quantitative analysis by real-time PCR

Total RNA was extracted from the ganglion-like structure of organotypic cultures as described above. cDNA was amplified in PCR solution containing 20 μL of Power SYBR Green PCR Master Mix (Thermo Fisher Scientific) as previously described,^3^^,^^18^ with synthesized primers targeting *Atf3* (sense: 5′-AGA GCT GAG ATT CGC CAT CC-3′, antisense: 5′-GTT TCG ACA CTT GGC AGC AG-3′, GenBank: NM_012912.2), or *Il6* (sense: 5′-TTC CAG CCA GTT GCC TTC TT-3′, antisense: 5′-TCA GAA TTG CCA TTG CAC AAC-3′, GenBank: NM_012589.2). PCR was performed using the StepOnePlus System (Thermo Fisher Scientific). GAPDH (primers shown in Table S3) was used as a normalization control to calculate relative mRNA levels.

#### Infection of AAV into the organotypic cultures

At 20 days after seeding, the organotypic cultures were exposed to AAV-hSyn-GCaMP6m diluted 1:100 in the culture medium for 2 days. After incubation, medium containing AAV was washed out and replaced with the fresh culture medium. The following Ca^2+^ imaging experiments were conducted a week after infection.

#### Ca^2+^ imaging analysis

At least 1 h before imaging, the culture media were replaced with a 60 μL volume of media in each target chamber. The GCaMP6m fluorescence was recorded and analyzed using a time-lapse image analysis system (HCImage; Hamamatsu Photonics, Shizuoka, Japan) equipped with a digital camera (ORCA-Flash 4.0, Hamamatsu Photonics) and a fluorescence microscope (ECLIPSE Ti-E; Nikon). During experiments, organotypic cultures were placed in a stage top incubator of the system, and the environment in the incubator was generally held to be a temperature of 37°C with gas concentration of 5% CO_2_. The fluorescence images in the neuronal cell bodies of the ganglion-like structure were acquired every 1 s per frame using a 10× objective lens (Nikon). As shown in Figures 3A and 40 μL droplets of the media containing KCl (final conc. 10 or 30 mM), capsaicin (final conc. 1 μM), or AITC (final conc. 300 μM) were applied onto the nerve endings in the targeting chamber at 0 s. In experiments examining suppression of cell excitability by lidocaine, the culture medium was replaced with lidocaine (3 mM)-containing medium 1 h before KCl treatment. To obtain *ΔF/F0*, *F0* was calculated by averaging the fluorescence intensity data of each cell at 3 time points just before KCl application. *ΔF/F0* was calculated from randomly chosen approximately 30 neuronal cell bodies within the ganglion of each organotypic culture, and its average values were indicated as dotted traces in Figures 3C–3G. The mean *ΔF/F0* of 6 (Figure 3C), 5 (Figure 3D), 3 (Figures 3E and 3F) or 4 (Figure 3G) organotypic cultures is indicated as bold traces.

#### Transection of the axon bundles

The axon bundles of the organotypic cultures were transected at the middle of the microchannel using a 27G needle around 28 days after DRG seeding as shown in Figure 4A. The debris were removed from the chambers using micro forceps soon after transection. After transection, culture media was replaced every 2–3 days. The bright field images of the organotypic cultures shown in Figure 4D and images shown in Figure S4B were obtained using MuviCyte (Revvity, Waltham, MA, USA). The regenerated nerve axons were collected for immunohistochemistry and electron microscopy 14 days after transection.

### Quantification and statistical analysis

#### Statistical analysis

Data were analyzed using GraphPad Prism 8 (GraphPad Software, San Diego, CA, USA) and expressed as means ± S.E.M. Differences between two groups were compared using unpaired t test (Figure 4B) or Mann-Whitney U-test (Figure 4C). In all cases, *p < 0.05* was considered statistically significant.

## Supplemental information

Document S1. Figures S1–S4 and Tables S1–S3

Document S2. Article plus supplemental information
