# Supplementary material for: Development of a 3-dimensional organotypic model with characteristics of peripheral sensory nerves
Source: Cell Rep Methods. 2024 Aug 7;4(8):100835. doi: 10.1016/j.crmeth.2024.100835 (PMC11384078; doi:10.1016/j.crmeth.2024.100835)
Supplement: Document S1. Figures S1–S4 and Tables S1–S3 [file mmc1.pdf]

Cell Reports Methods, Volume *4*

Supplemental information

Development of a 3-dimensional organotypic model with characteristics of peripheral sensory nerves

Madoka Koyanagi, Ryosuke Ogido, Akari Moriya, Mamiko Saigo, Satoshi Ihida, Tomoko Teranishi, Jiro Kawada, Tatsuya Katsuno, Kazuo Matsubara, Tomohiro Terada, Akira Yamashita, and Satoshi Imai

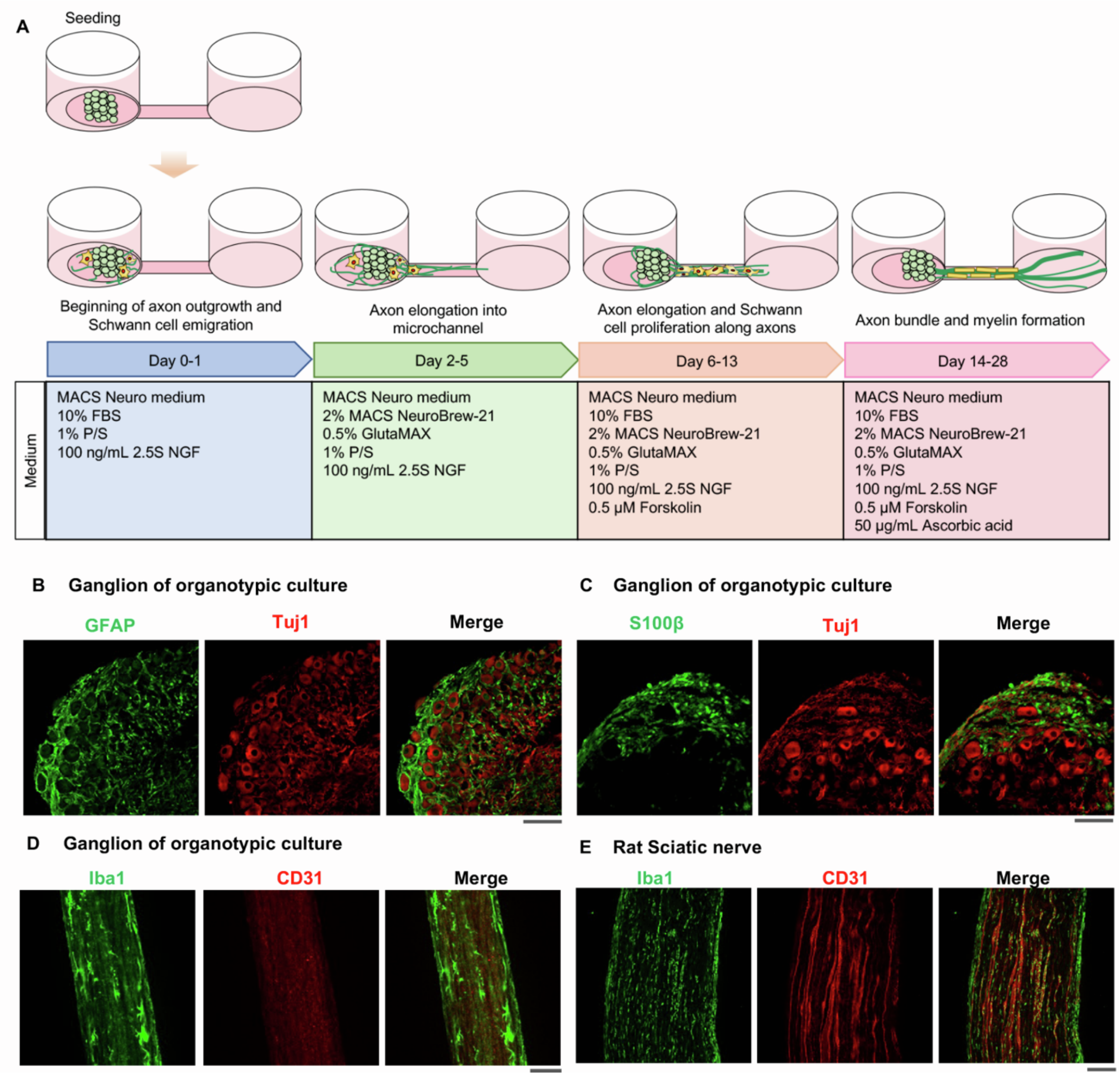

Supplemental Figure S1. Details for the organotypic culture development, and existence of glial cells and macrophages in the organotypic culture, related to Figure 1. (A) Schematic illustration of the organotypic culture development on the organoid culturing chamber and medium composition over the culture period. An axon bundle with myelin is gradually formed in the microchannel of the microchip after seeding DRG explant obtained from E15.5 rat embryo. (B) Representative confocal images showing GFAP-positive satellite glia cells (green) surrounding Tuj1-positive neuronal cell bodies (red) in the ganglion-like structure of the organotypic culture 28 days after seeding. (C) Representative confocal images showing S100β-positive Schwann cells (green) found around Tuj1-positive neuronal cell bodies (red) in the ganglion-like structure of the organotypic culture 28 days after seeding. (D) Representative confocal images showing Iba1-positive cells (putative residential macrophages, green) and CD31-positive blood vessels (red) in the axon bundle of the organotypic culture 28 days after seeding. CD31-positive blood vessels were not identified in the axon bundles. (E) Staining pattern of Iba1 (green) and CD31 (red) in the rat sciatic nerve. The same antibodies used in (D) were used. For staining of rat sciatic nerves, male

Wistar/ST rats (4 weeks old) were deeply anesthetized with isoflurane and intracardially perfused with 4% PFA in 0.1 M PB. After perfusion, sciatic nerves were quickly removed. Sciatic nerves were post-fixed in 4% PFA for 4 h and permeated with 15% sucrose solution in 0.1 M PB for 24 h at 4°C. The sections were frozen in an embedding compound. Frozen longitudinal segments of sciatic nerve were cut with a freezing cryostat (Leica CM 1850, Leica Microsystems) (16 µm thick) and thaw-mounted on MAS-coated glass slides (Matsunami Glass). Scale bars: 50 µm (B–D), 200 µm (E).

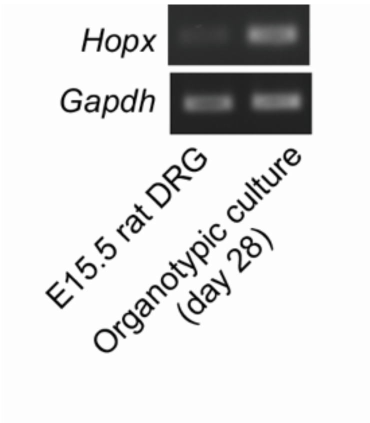

Supplemental Figure S2. Difference in the expression of *Hopx* mRNA between E15.5 rat DRG and organotypic culture 28 days after seeding, related to Figure 2. Representative RT-PCR bands showing mRNA levels of *Hopx*, a transcriptional factor that is known to increase its expression after sensory neuron differentiation, in the E15.5 rat DRG (*left lane*) and ganglia of organotypic culture 28 days after seeding (*right lane*). To amplify *Hopx*, the following synthesized primers were used; sense: 5'-TCA ACA AGG TCA ACA AGC AC-3', antisense: 5'-GCG CTG CTT AAA CCA TTT CT-3' (GenBank^{TM}: NM_133621.3). *Gapdh* was used as an internal control.

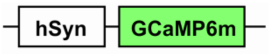

Supplemental Figure S3. Illustration of AAV-hSyn-GCaMP6m construct, related to Figure 3. AAV bearing *GCaMP6m* gene under the control of the 476-bp human Synapsin (hSyn) promoter were used.

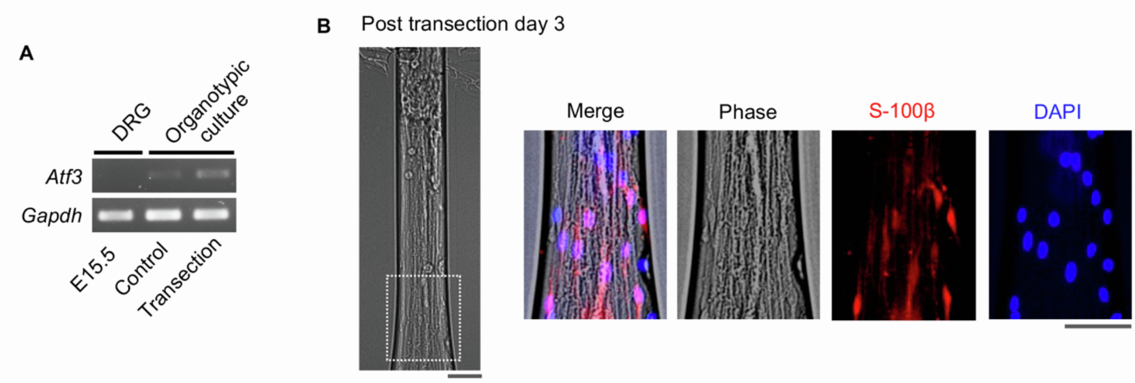

Supplemental Figure S4. Changes after axonal transection of the organotypic culture, related to Figure 4. (A) Representative RT-PCR bands showing expression of *Atf3* mRNA in E15.5 rat DRG (*left lane*) and the ganglia of mature organotypic cultures without transection (Control; *middle lane*) or 1 day after transection (Transection; *right lane*). *Gapdh* was used as an internal control. (B) Representative images showing migrating S100β-positive Schwann cells (red) along regenerated nerve fibers 3 days after axonal transection of the organotypic culture. Scale bars: 50 μm.

Target name	Species		Primer sequence	GenBank^{TM} sequence accession number
Scn9a	Rat	Sense	5'-TAT CCT GTT CAC TGG GGA GT-3'	NM_133289.2
		Antisense	5'-TAG GGT CAC AAT CAG GAG GT-3'	
Scn10a	Rat	Sense	5'-AAA CGC CAA TGA GAG AAG AC-3'	NM_ 017247.2
		Antisense	5'-TCA AGC TCC TCA ATG ACA GA-3'	
Kcna1	Rat	Sense	5'-TTC ATT GTG GAA ACC TTG TG-3'	NM_173095.3
		Antisense	5'-GCC AAT GAA GAG GAA AAA GA-3'	
Kcna2	Rat	Sense	5'-CCC TCA AGA CAC CTA TGA CC-3'	NM_012970.4
		Antisense	5'-AAA ATG GCA TCA AAG CTA GG-3'	
Kcna4	Rat	Sense	5'-ACC TAC TGC CAC AGG ATG AT-3'	NM_012971.3
		Antisense	5'-TGA CCA GGA CAG ATA CGA TG-3'	
Kcnk2	Rat	Sense	5'-CGG AAG CTC TTT CTT CTT TG-3'	NM_172042.2
		Antisense	5'-TGA AGA TGA TGG TCG AGA TG-3'	
Kcnk4	Rat	Sense	5'-GGG ACC ATC ATC ACT ACC AT-3'	NM_053804.2
		Antisense	5'-ATA ATC GCC AAA GCC TAC AG-3'	
Cacna1b	Rat	Sense	5'-GAC AGT GGG GAA GGT CTA TG-3'	NM_001195199.1
		Antisense	5'-ATG TCA TGT TCT GCA TCT GG-3'	
Cacna1h	Rat	Sense	5'-GGG TGA ACA TGC CTA CCT AC-3'	NM_153814.2
		Antisense	5'-TCC TTA GAT GAC AGC ACG AA-3'	
Cacna2d1	Rat	Sense	5'-ATC GAA GAC GCT AAT TTT GG-3'	NM_012919.3
		Antisense	5'-ACA GAT GTC CGG ATG AGT TT-3'	

Supplemental Table S1. Sequences of synthesized primers used in RT-PCR, related to Figure 2G.

Target name	Species		Primer sequence	GenBank™ sequence accession number
Trpa1	Rat	Sense	5'-ATG TTC GTC CCA ATT GTT CT-3'	NM_207608.1
		Antisense	5'-CTT CAG CCG GTA TTT CTG TT-3'	
Trpv1	Rat	Sense	5'-TGG AGA ATG GAG CAG ATG TC-3'	NM_031982
		Antisense	5'-GGA CAG GTC ATA AAG GGA GG-3'	
Trpm8	Rat	Sense	5'-AAC AAT CAT ACC CAC CTG CT-3'	NM_ 134371.3
		Antisense	5'-AAC ATC CTC TAC CTC CAC CA-3'	
Ntrk1	Rat	Sense	5'-TTG CTC ATG GTC TTC GAG TA-3'	NM_ 021589.1
		Antisense	5'-CTG AAC TTG CGG TAG AGG AT-3'	
Ntrk2	Rat	Sense	5'-GGG AAG TCT GTG ACC ATT TC-3'	NM_ 012731.3
		Antisense	5'-CGT CCT TCC CAT ATT CAT TC-3'	
P2rx3	Rat	Sense	5'-CCA GCT GCT GAT TAT CTC CT-3'	NM_031075.2
		Antisense	5'-CAT CAT GAT AGG CAT CTC CA-3'	
Asic1	Rat	Sense	5'-CGA CTC CTA CAG CAT CAC TG-3'	NM_024154.2
		Antisense	5'-AGC CTG TGC TTA ATG ACC TC-3'	
Npy2r	Rat	Sense	5'-TGC AAG TGA CCA TTA CCA TC-3'	NM_ 023968.1
		Antisense	5'-TAA TGT GGA ACA CGG TGA AG-3'	

Supplemental Table S2. Sequences of synthesized primers used in RT-PCR, related to Figure 2H.

Target name	Species		Primer sequence	GenBank™ sequence accession number
Bdkrb2	Rat	Sense	5'-ATC ACC ATC GCC AAT AAC TT-3'	NM_001270713.2
		Antisense	5'-CCC TGT AGT CCT TCA TGG TC-3'	
Hrh1	Rat	Sense	5'-CTA CCT CCC CAC TTT GCT TA-3'	NM_017018.2
		Antisense	5'-CCG ACA AGT ATT CAG GCT CT-3'	
Ptger4	Rat	Sense	5'-ATT CCC GCA GTG ATG TTT AT-3'	NM_032076.3
		Antisense	5'-GCC GAA GAA AAG TAG GAT GA-3'	
Adora2a	Rat	Sense	5'-CAA CAG TAA CCT GCA GAA CG-3'	NM_053294.5
		Antisense	5'-GCC AGA AAA ATC CGT AGG TA-3'	
Il1r1	Rat	Sense	5'-AAT GCA CGG AGT ATC CAA AT-3'	NM_013123.3
		Antisense	5'-CTT CCC CTG GTA TGT GTA GG-3'	
Oprm1	Rat	Sense	5'-GGC TGG TCC ATG TAA GAA TC-3'	NM_013071.2
		Antisense	5'-TAG AGG GCC ATG ATG GTA AT-3'	
Cx3cl1	Rat	Sense	5'-GTA GAG GAA CCC ATT CAT GC-3'	NM_134455.2
		Antisense	5'-CGT ACG AGT TAC TGC CAC AG-3'	
Il1b	Rat	Sense	5'-TTC AAA TCT CAC AGC AGC AT-3'	NM_031512.2
		Antisense	5'-GGG AAC ATC ACA CAC TAG CA-3'	
Il6	Rat	Sense	5'-AAT CTG CTC TGG TCT TCT GG-3'	NM_012589.2
		Antisense	5'-GCC ACT CCT TCT GTG ACT CT-3'	
Ccl2	Rat	Sense	5'-TTG TCA CCA AGC TCA AGA GA-3'	NM_031530.1
		Antisense	5'-GGT TGT GGA AAA GAG AGT GG-3'	
Ccl7	Rat	Sense	5'-CCA CTT GCT GCT ATG TCA AG-3'	NM_001007612.1
		Antisense	5'-AGG GCT TTG GAG TTG AAG TT-3'	
Tnfa	Rat	Sense	5'-ATG ATC CGA GAT GTG GAA CT-3'	NM_012675.3
		Antisense	5'-ACC ACC AGT TGG TTG TCT TT-3'	
Gapdh	Rat	Sense	5'-GTT ACC AGG GCT GCC TTC TC-3'	NM_017008
		Antisense	5'-TGA TGA CCA GCT TCC CAT TC-3'	

Supplemental Table S3. Sequences of synthesized primers used in RT-PCR, related to Figure 2I.
